# Supplementary material for: Coreleased Orexin and Glutamate Evoke Nonredundant Spike Outputs and Computations in Histamine Neurons
Source: Cell Rep. 2014 Apr 24;7(3):697–704. doi: 10.1016/j.celrep.2014.03.055 (PMC4022832; doi:10.1016/j.celrep.2014.03.055)
Supplement: Document S1. Supplemental Experimental Procedures and Figure S1 [file mmc1.pdf]

Cell Reports, Volume *7*

Supplemental Information

Coreleased Orexin and Glutamate Evoke Nonredundant Spike Outputs and Computations in Histamine Neurons

Cornelia Schöne, John Apergis-Schoute, Takeshi Sakurai, Antoine Adamantidis, and Denis Burdakov

SUPPLEMENTARY MATERIALS AND METHODS

1. Genetic targeting and viral transduction

Animal procedures were in accordance with UK Home Office regulations. Expression of ChR2 in OHNs was carried out and functionally confirmed as illustrated in Fig. S1. Briefly, high-titer ($1.5*10^{12}$ vg/ml) adeno-associated viral (AAV) constructs rAAV2/1.EF1a-DIO-hChR2(H134R)-EYFP.WPRE.hGH (Addgene #20298; packaged at Vector Core, University of Pennsylvania) were stereotaxically injected into brains of orexin-Cre mice, bred in het-WT breeding pairs with C57BL/6 mice. 4-6 week-old male and female mice were anesthetized with isoflourane and head-fixed in a stereotaxic frame (David Kopf Instruments). In some experiments in Fig. 3A,B, we also used rAAV2/Ef1a-DIO-ChR2(E123T/T159C)-mcherry ($3*10^{12}$ vg/ml; Addgene #35510 packaged at Vector Core, University of Pennsylvania) with similar results (data from two AAVs were pooled in this figure).

A borosilicate glass pipette tip (20-40 μm diameter) was stereotaxically lowered into the lateral hypothalamus. Three injections (each 50 nl, delivered at 75 nl/min) were made into LH in each hemisphere (bregma: -1.3 to -1.4 mm; midline ±0.9 mm; dorsal surface: -5.30, -5.15, and -5.00 mm). The pipette was gently withdrawn 6 min after final injection. Injections of identical AAV constructs into the lateral hypothalamus of WT mice did not generate ChR2-eYFP expression in the lateral hypothalamus (n = 3 mice), confirming that ChR2 expression was specific to Cre-containing cells.

2. Immunolabelling

We tested for specificity of transgene expression in orexin-Cre mice using immunohistochemistry. Orexin-Cre mice that had been previously injected with AAV containing ChR2-eYFP were terminally anesthetized and transcardially perfused with phosphate buffered saline containing 4 % PFA. Following postfixation brains were sectioned into 50 μm thick slices using a cryostat. Slices were stained using rabbit anti orexin-A (Phoenix Pharmaceutics, Inc., USA; 1:250) and mouse anti Cre-recombinase (Millipore, USA; 1:500) antibodies. We used goat anti rabbit alexa 647 (1:500) and goat anti mouse alexa 488 (1:1000) from Invitrogen for fluorescent labelling.

To label HANs during whole-cell recordings, these were filled with 0.2 % biocytin (Tocris) that was added to the intracellular recording solution. Cells were kept in the whole-cell mode for a minimum of 20 min. After recovery for at least 20 min, the tissue was fixed in 4 % PBS overnight. Biocytin filled cells were labeled with rabbit anti adenosine deaminase (Chemicon; 1:250). Immunofluorescence was achieved using goat anti-rabbit Alexa 555 (Invitrogen; 1:1000) antibodies and streptavidin tagged with Cy2 (Invitrogen; 1:1000).

Images were taken using an Olympus BX61WI confocal microscope (Olympus FluoView v 2.1b software) in a dynamic range of 16 bit using a 25x water immersion objective (NA 1.05, Olympus). Cy2 was excited with an argon laser at 488 nm, and its fluorescence collected between 570 and 670 nm using a spectral detector (Olympus). Alexa 555 was excited with a diode-pumped solid-state (DPSS) laser at 559 nm, and fluorescence emission collected at 570-670 nm using a spectral detector (Olympus).

When more than one fluorophore was detected, the sequential “between-lines” scanning mode of the microscope was used to achieve optimal separation of fluorescent signals.

3. Photostimulation and electrophysiology

Coronal slices containing the tuberomammillary hypothalamic nucleus (TMN) were made ≈2 months post-injection. 250 μm thick slices were cut with a Leica VT 1200S vibratome in ice-cold ACSF (see below), and allowed to recover for 1 hour at 35 °C in ACSF before recordings. For ChR2 stimulation with blue light, we used a LAMBDA DG-4 fast beam switcher (Sutter Instruments) with a Xenon lamp and ET470/40 nm band pass filter. Flashes of light (5 ms duration, ~10 mW/mm^2 power) were delivered onto ChR2-containing axons around the recorded cell via a 40x objective with 0.8 NA. Patch pipettes were manufactured from borosilicate glass, and their tip resistances were 4-6 MΩ when filled with K-gluconate solution (see below). Whole-cell recordings were carried out at 37 °C using an EPC-10 amplifier and PatchMaster software (HEKA Elektronik, Germany). Orexin/hypocretin neurons (identified by eYFP fluorescence and confirmed by electrical fingerprinting) routinely followed long trains of light flashes with time-locked trains of action potentials (Fig. S1A). Histamine neurons in the ventral TMN were unambiguously identified using classical electrophysiological, morphological, and post-immunostaining criteria (described with full references to original papers in Schöne et al., J Neurosci 2012, 32: 12437-12443). To dissect contributions of orexin/hypocretin and glutamate, we compared histamine neuron responses with and without pharmacological blockers of different neurotransmitter receptors. Only cells with access resistances of <20 MΩ were used for analysis. Current signals were low-pass filtered at 3 kHz and digitized

at 10 kHz. Glutamate EPSCs were identified and analyzed using standard Minianalysis software (Synaptosoft). Only histamine neurons that showed fast synaptic responses to orexin/hypocretin neuron optical stimulation were used for further analysis.

Control experiments revealed that basal tone (without optical stimulation) of orexin/hypocretin or glutamate was too low to drive histamine neuron firing. Specifically, blocking OH2Rs with TCS did not alter spontaneous histamine neuron firing, as analyzed by examining 10 s intervals preceding optical stimulation (control: 1.7±1.0 Hz, TCS: 1.6±0.7 Hz, n=4 cells, p>0.5 by paired t-test). TCS also did not significantly shift baseline current at -70 mV (control: -38.36±7.1 pA, TCS: -36.4±9.1 pA, n=5 cells, p>0.5 by paired t-test). Similarly, blocking OH2Rs+OH1Rs (with TCS+SB, see Methods) did not alter spontaneous histamine neuron firing (control: 2.2±1.0 Hz, TCS+SB: 2±0.4 Hz, n=6 cells, p>0.5 by paired t-test). Finally, blocking AMPAR-mediated glutamate transmission with CNQX did not alter spontaneous histamine neuron firing (control: 2.7±0.7 Hz, CNQX: 2.4±0.8 Hz, n=6 cells, p>0.3 by paired t-test). CNQX also did not significantly shift baseline current at -70 mV (control: -25.8±5.7 pA, TCS: -23.7±3.6 pA, n=5 cells, p>0.4 by paired t-test).

4. Analysis

Data were analysed with Minianalysis (Synaptosoft), Matlab (Mathworks) or Prism (Graphpad). Histamine neuron firing responses to optical orexin neuron stimulation were separated from baseline firing by subtracting the average baseline firing during 10 s prior to stimulation in each cell. In group data plots, results are presented as means ± sem of multiple experimental trials (1-2 trials were recorded per cell), except

where stated otherwise. Student's unpaired or paired t-test, or one or two-way ANOVA followed by Bonferroni or Newman-Keuls posthoc tests, were used for statistical hypothesis testing; the significance labels in the figures are *$p < 0.05$, **$p < 0.01$, ***$p < 0.001$, and $p > 0.05$ was taken as non-significant (ns).

5. Chemicals and solutions

ACSF was bubbled with 95% O_2 and 5% CO_2 and contained (in mM): 125 NaCl, 2.5 KCl, 2 $MgCl_2$, 2 $CaCl_2$, 1.2 NaH_2PO_4, 21 $NaHCO_3$, 1 d-(+)-glucose, 0.1 Na^+-pyruvate and 0.4 ascorbic acid. For whole-cell recordings, pipettes were filled with (in mM): 124 K-gluconate, 14 KCl, 10 HEPES, 1 EGTA, 5 MgATP, 0.3 Na_2GTP, 10 Na_2Phosphocreatine, pH = 7.3 with KOH. Orexin/hypocretin receptors were blocked with either 10 μM TCS-OX2-29 ("TCS", an OHR2 receptor blocker), and/or 10 μM SB-334867 ("SB", an OHR1 blocker at this concentration). AMPA glutamate receptors were blocked with 10 μM 6-cyano-7-nitroquinoxaline-2,3-dione (CNQX). NMDA glutamate receptors were blocked with 50 μM (2*R*)-amino-5-phosphonovaleric acid (AP5). $GABA_{A/C}$ receptors were blocked with 50 μM picrotoxin (PiTX). For bath application of orexin/hypocretin, we used 300 nM orexin-A from Bachem. All other chemicals were from Sigma or Tocris.

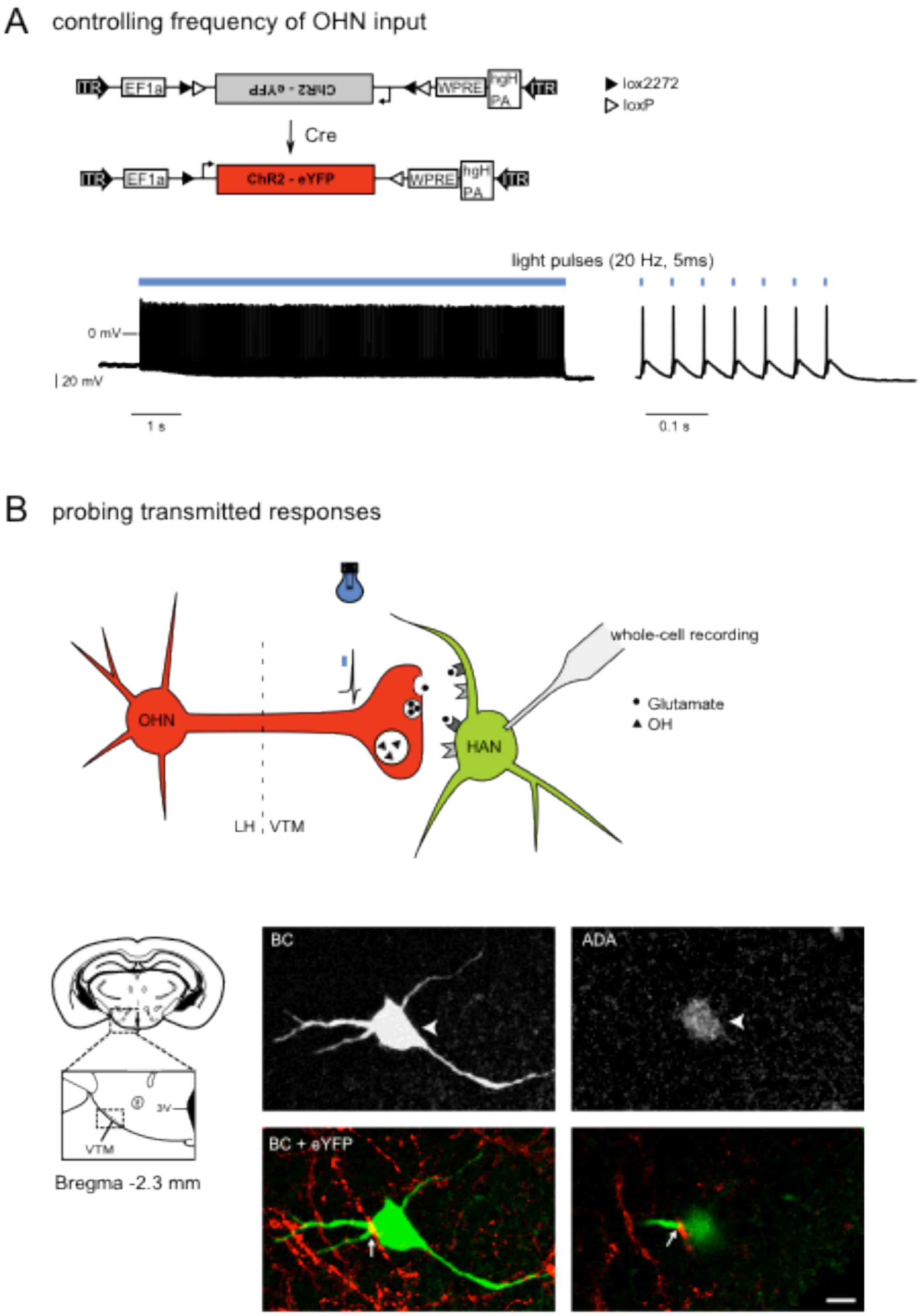

Figure S1. *Studying functional transmission in orexin → histamine microcircuit* (Illustration of methodological background to experiments in main Figures 1-3).

A. Schematic of virally-delivered constructs used to transduce orexin-Cre neurons (OHNs) with ChR2 (top), after which OHN spikes routinely followed optical stimuli in brain slices (bottom, typical example of n = 10 cells).

B: Schematic of experimental strategy for probing postsynaptic responses of histamine neurons (HANs) to OHN stimulation (top), and typical confocal images (bottom) of a HAN filled with biocytin (BC) and confirmed by post-recording immunocytochemistry to contain HAN marker adenosine deaminase (ADA, arrowheads, n = 3/3 cells). ChR2-eYFP fibers (shown in red pseudo-color) make close contact with HAN (arrows in bottom images). Scale bar, 10 µm.
