# Supplementary material for: Coreleased Orexin and Glutamate Evoke Nonredundant Spike Outputs and Computations in Histamine Neurons
Source: Cell Rep. 2014 Apr 24;7(3):697–704. doi: 10.1016/j.celrep.2014.03.055 (PMC4022832; doi:10.1016/j.celrep.2014.03.055)
Supplement: Document S2. Article plus Supplemental Information [file mmc2.pdf]

Cell Reports
Report

OPEN ACCESS
CellPress

Coreleased Orexin and Glutamate Evoke Nonredundant Spike Outputs and Computations in Histamine Neurons

Cornelia Schöne,[1] John Apergis-Schoute,[2] Takeshi Sakurai,[3,4] Antoine Adamantidis,[5,6] and Denis Burdakov[1,7,*]
[1]Division of Neurophysiology, MRC National Institute for Medical Research, London NW7 1AA, UK
[2]Department of Pharmacology, University of Cambridge, Cambridge CB2 1PD, UK
[3]Department of Molecular Neuroscience and Integrative Physiology, Faculty of Medicine, Kanazawa University, Kanazawa, Ishikawa 920-8640, Japan
[4]International Institute for Integrative Sleep Medicine, University of Tsukuba, Tsukuba, Ibaraki 305-8575, Japan
[5]Neurology Department, Bern University Hospital, 3010 Bern, Switzerland
[6]Department of Psychiatry, McGill University, Montreal, QC H3A 0G4, Canada
[7]MRC Centre for Developmental Neurobiology, King's College London, London WC2R 2LS, UK
*Correspondence: denis.burdakov@kcl.ac.uk
http://dx.doi.org/10.1016/j.celrep.2014.03.055
This is an open access article under the CC BY license (http://creativecommons.org/licenses/by/3.0/).

SUMMARY

Stable wakefulness requires orexin/hypocretin neurons (OHNs) and OHR2 receptors. OHNs sense diverse environmental cues and control arousal accordingly. For unknown reasons, OHNs contain multiple excitatory transmitters, including OH peptides and glutamate. To analyze their cotransmission within computational frameworks for control, we optogenetically stimulated OHNs and examined resulting outputs (spike patterns) in a downstream arousal regulator, the histamine neurons (HANs). OHR2s were essential for sustained HAN outputs. OHR2-dependent HAN output increased linearly during constant OHN input, suggesting that the OHN→HAN^{OHR2} module may function as an integral controller. OHN stimulation evoked OHR2-dependent slow postsynaptic currents, similar to midnanomolar OH concentrations. Conversely, glutamate-dependent output transiently communicated OHN input onset, peaking rapidly then decaying alongside OHN→HAN glutamate currents. Blocking glutamate-driven spiking did not affect OH-driven spiking and vice versa, suggesting isolation (low cross-modulation) of outputs. Therefore, in arousal regulators, cotransmitters may translate distinct features of OHN activity into parallel, nonredundant control signals for downstream effectors.

INTRODUCTION

During chemical communication between neurons, transmitters released by presynaptic activity evoke long-range postsynaptic signals (action potentials, spikes). Small transmitters made in presynaptic terminals (e.g., glutamate, GABA, ATP, acetylcholine) are recognized regulators of postsynaptic spiking (Bear et al., 2001; Ren et al., 2011). They can be coreleased with larger neuropeptides, which are encoded by the genome, highly diverse, and widely present in central terminals (Bear et al., 2001; Burnstock, 2004; Salio et al., 2006; Burbach, 2011). However, in the brain, knowledge of spike patterns arising from activity-dependent neuropeptide release from defined neurons remains imperfect.

To study input-output computations and spike patterns resulting from neural release of a behaviorally vital neuropeptide, we probed relations between pre- and postsynaptic activity in a brain microcircuit comprising orexin/hypocretin and histamine neurons. Orexin/hypocretin neuropeptides (OH) are critical for stable wakefulness, reward-seeking, and energy balance (de Lecea et al., 2006; Sakurai, 2007). OH-expressing neurons (OHNs) are located in the hypothalamus, project widely throughout the brain (Peyron et al., 1998), and are activated by diverse environmental challenges such as sensory stimuli (e.g., sounds), fasting, hypoglycemia, hypercapnia, and stress (Mileykovskiy et al., 2005; Sakurai, 2007; Sakurai et al., 1998; Williams et al., 2007, 2008; Winsky-Sommerer et al., 2004). OHN firing promotes awakening in a frequency-dependent manner (Adamantidis et al., 2007), while OHN loss causes narcolepsy (Hara et al., 2001; Thannickal et al., 2000; Mignot et al., 2002; Ripley et al., 2001). Narcolepsy also results from lack of OH peptides or OH type-2 G protein coupled receptors (OHR2), emphasizing the importance of OH signaling (Chemelli et al., 1999; Lin et al., 1999; Peyron et al., 2000; Willie et al., 2003). OH peptides are stored in dense-core vesicles, in the same terminals as clear vesicles associated with small transmitters (de Lecea et al., 1998). However, there is little direct evidence that OH peptides are released by OHN firing to evoke spiking in downstream targets. So far, the firing of OHNs has only been shown to release glutamate (Schöne et al., 2012). At the circuit level, the relative roles of OH and glutamate remain unclear.

To address this, we used optogenetics (Petreanu et al., 2007; Yizhar et al., 2011) to stimulate OHNs in situ. We measured resulting responses in wake-promoting histamine neurons

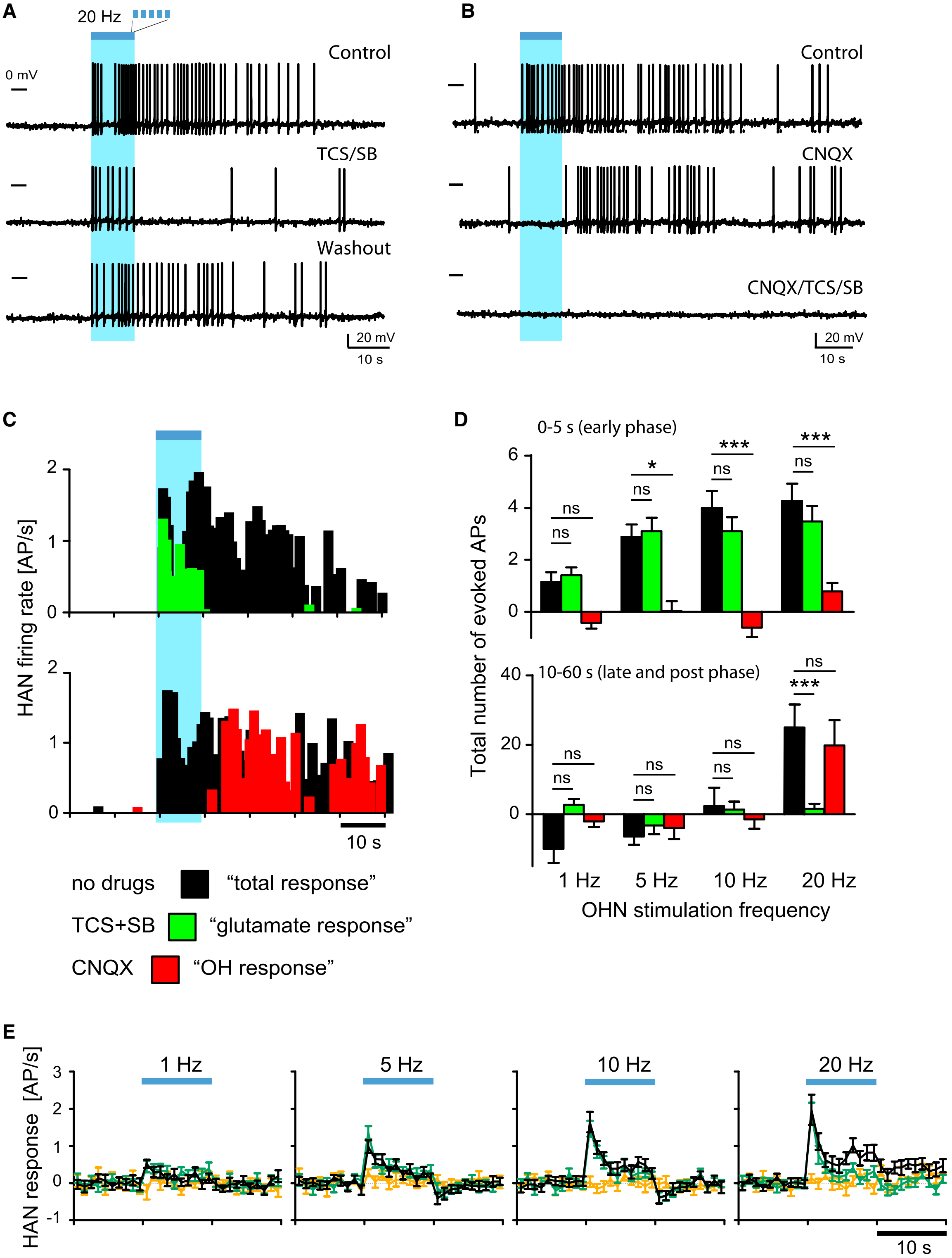

Figure 1. Dissociating OH and Glutamate Actions on HAN Output

(A and B) Examples of HAN spiking caused by OHN stimulation (blue bars = 20 Hz stimulation). (A) All three traces are from the same representative cell, showing the effect of OHR block (TCS/SB, n = 11 cells). (B) All three traces are from the same representative cell (different from A), showing the effect of AMPAR block (CNQX, n = 5 cells) and OHR + AMPAR block (CNQX/TCS/SB, n = 5 cells).

(C) Rate histograms for (A) and (B).

(legend continued on next page)

(HANs) of the tuberomammillary hypothalamus, one of the key postsynaptic targets of OHNs expressing the "antinarcoleptic" OHR2s (Yamanaka et al., 2002; Willie et al., 2003; Haas et al., 2008; Schöne et al., 2012). Wakefulness instability produced by global OHR2 deletion is reversed by local OHR2 rescue in tuberomammillary hypothalamus, consistent with the importance of this circuit for brain state control (Haas et al., 2008; Mochizuki et al., 2011; Sakurai, 2007). Simultaneous control of OHN input, pharmacological manipulation of transmission, and recording of HAN output enabled us to compare circuit performance and computational operations enabled by OH versus glutamate transmission.

RESULTS

Pharmacological Dissociation of Histaminergic Representations of OHN Activity

To study input-output computations in the OHN→HAN circuit, we optically triggered spikes in OHNs, and measured responses in HANs using intracellular patch-clamp recordings in mouse brain slices (Figure S1; Supplemental Experimental Procedures). Spontaneous OH or glutamate transmission was too low to affect HAN firing in our preparation (Supplemental Experimental Procedures, Section 3). To evoke transmission in the OHN→HAN circuit, we first drove OHNs with brief optical stimuli (10 s trains of flashes at 20 Hz), producing OHN spike bursts (Figure S1A) similar to those emitted by OHNs upon sensory stimulation in vivo (Mileykovskiy et al., 2005). This produced rapid postsynaptic excitation in ~70% of HANs (Figure 1; n = 116/173 cells). Continuing to analyze HAN firing pattern after high-frequency stimulation revealed a late excitation (Figures 1A–1D), blocked by a mixture of OH receptor antagonists (SB + TCS; Figures 1A–1D). In the same cells, blocking glutamate AMPA receptors (AMPARs) with CNQX abolished only rapid excitation during the 10 s of stimulation (Figures 1B–1E).

Direct (versus modulatory) control of neural firing has been discussed as a relatively minor action of naturally released neuropeptides (Salio et al., 2006; van den Pol, 2003; Schöne and Burdakov, 2012). However, for the same OHN stimulation (20 Hz for 10 s), we estimated that OH transmission generated ~5-fold more spikes than glutamate (during 0–60 s relative to the stimulation; in CNQX: 25.2 ± 6.6 spikes, n = 16 versus in SB + TCS: 5.1 ± 0.9 spikes, n = 26; $p < 0.001$ by unpaired t test; see also Figure 1D). This suggests that OH and AMPARs may generate distinct, temporarily and pharmacologically separable spike patterns. The difference in speeds of the two spike responses presumably relates, in part, to the transmitters' actions on the slow metabotropic OHRs versus fast ionotropic AMPARs (Schöne et al., 2012; Sakurai, 2007).

Frequency Dependence of Histaminergic Representations of OHN Activity

Studies using nonselective stimulation (high potassium, electric shocks) and unphysiological detection (e.g., radioimmunoassay) suggested that neuropeptide release required more stimulation than the release of smaller transmitters (Dutton and Dyball, 1979; Haass et al., 1989; Lundberg et al., 1986; Verhage et al., 1991). However, physiological interpretation of this could be confounded by (1) possible stimulation of off-target neurons/axons, glial, endothelia, etc.; or (2) lower detection sensitivity than may exist in intrinsic detectors, leading to release underestimates. We re-examined stimulation requirements for neuropeptide action using the selective stimulation and intrinsic detection in the OHN→HAN circuit across OHN activity linked to behavior in vivo (1–20 Hz firing, Adamantidis et al., 2007; Lee et al., 2005; Mileykovskiy et al., 2005). We observed OH-dependent excitation only at upper frequencies and glutamate-dependent excitation across all frequencies (Figure 1D).

Synaptic inputs can be modulated by artificially (bath) applied OH peptides (e.g., Lambe and Aghajanian, 2003; Ma et al., 2007; van den Pol et al., 1998). We thus examined interactions between OH and glutamate-dependent firing across stimulation frequencies. At every frequency, glutamate-dependent firing was unaffected by blockers that abolished OH-dependent firing, and vice versa (Figures 1D and 1E). OHNs also contain chemical markers for other transmitters (Crocker et al., 2005; Furutani et al., 2013). However, coapplication of OH and glutamate receptor blockers abolished the effects of OHN stimulation on HAN firing (Figures 1B and 1E; evoked spikes at 20 Hz stimulation = 0.7 ± 5, n = 5 cells, $p > 0.2$, one-sample t test).

This suggests that OH and glutamate are main drivers of long-range output in the OHN→HAN circuit and that OH transmission requires a higher presynaptic activity than glutamate transmission.

Temporal Relations between OHN Input and its Histaminergic Representations

Combining rapid reactions to stimulus trends with actions based on longer stimulus histories is useful for the brain, and for control systems in general (DiStefano et al., 2012). To explore how HANs fire during prolonged input, we extended OHN stimulation to 30 s. This roughly mimics in vivo OHN firing during behavioral transitions (e.g., from sleep to wakefulness) or during initiation of food consumption (Lee et al., 2005; Mileykovskiy et al., 2005). Prolonged OHN stimulation evoked two firing phases in HANs: a fast transient firing peak (similar to short stimulation, Figure 1E) followed by slow firing escalation overlapping in time with OHN stimulation (Figure 2A). OHR2 blockade (with 10 μM TCS, expected to block OXR2, but not OXR1; Hirose et al., 2003; Woldan-Tambor et al., 2011) abolished the slow phase but did not affect the fast, CNQX-sensitive phase (Figures 2A and 2B). In turn, the OHR2-dependent phase was unaffected by blockers of glutamate NMDA, AMPA, and $GABA_{A/C}$ receptors (Figure 2A). This further suggests that $OHN \rightarrow HAN^{OHR2}$ and $OHN \rightarrow HAN^{AMPAR}$ signaling modules control HAN output independently of each other and in complementary time domains.

(D) Evoked spikes (action potentials [APs]) versus stimulation frequency. Color-coding as in (C). Data are means ± SEM; cells for 1, 5, 10, and 20 Hz, respectively: control: n = 11, 14, 13, and 13; SB/TCS: n = 9, 12, 12, and 11; CNQX: n = 3, 3, 3, and 4.

(E) Temporal profiles of HAN firing responses across OHN stimulation frequencies. No drugs (black), SB/TCS (green), SB/TCS/CNQX (orange). Data are means ± SEM; cells for 1, 5, 10, and 20 Hz, respectively: control: n = 9, 12, 11, and 10; SB/TCS: n = 8, 11, 11, and 10; SB/TCS/CNQX: n = 5, 8, 8, and 7).

OPEN
ACCESS
CellPress

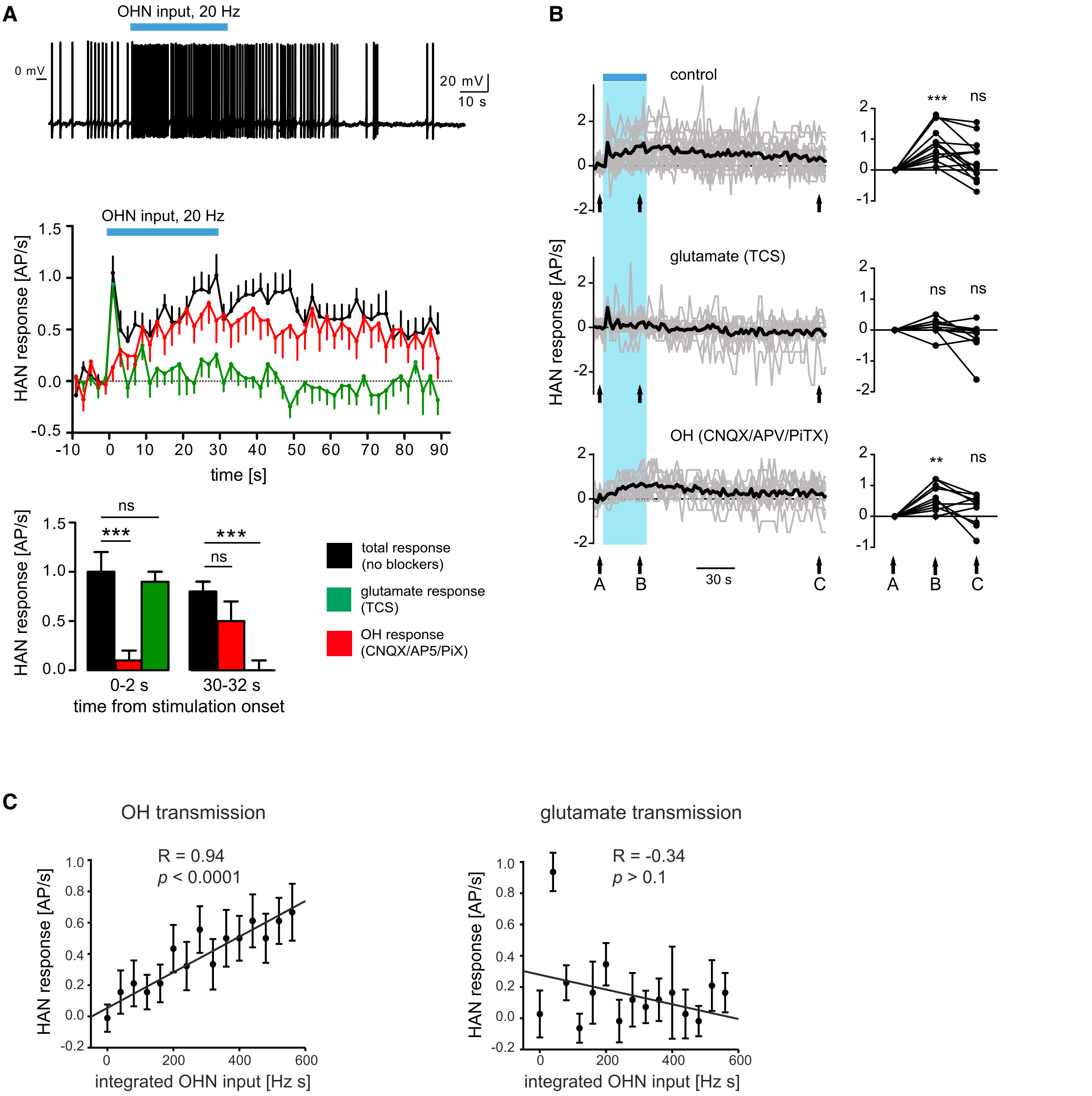

Figure 2. Time Courses of HAN Output in Relation to OHN Input

(A) Example (top trace, n = 17 cells) and group data (middle graph, means ± SEM) of HAN firing response to 30 s OHN stimulation (throughout the figure, blue bars = 20 Hz stimulation). Bottom: same data compared at specific time points.

(B) Same as (A) but on longer timescale to illustrate recovery and individual variations (means in black, individual cells in gray). Statistical comparisons are relative to baseline (arrow A).

(C) Relations between cumulative OHN input and HAN output during OH transmission (left graph, measured in CNQX/AP5/PiX) or glutamate transmission (right graph, measured in TCS). OHN input was 20 Hz for 30 s. R and p are linear regression fit parameters. Data are means ± SEM.

We next asked what features of OHN input are conveyed by the two HAN firing outputs. Amplitudes of glutamate-dependent output (shown in Figure 1E, green traces, recorded in OH blockers) correlated significantly with OHN stimulation frequency (one-way ANOVA, $F[3,36] = 3.45$, $p < 0.05$). However, decay time constants of the glutamate-dependent output did not vary significantly with OHN stimulation frequency (Figure 1E; extra sum-of-squares F test, $F[3,778] = 0.648$, $p > 0.05$, based on monoexponential decay fits). The OH-dependent output (which we observed and studied only at high OHN frequency) continued to rise throughout the 30 s of OHN input (Figure 2A, middle). There was a strong linear correlation between HAN output and cumulative (integrated) OHN input during this time (Figure 2C). When OHR2s were blocked, AMPARs could not maintain this input-output relation (Figure 2C).

This suggests that OH and glutamate drive distinct temporal patterns of HAN output and may thus be required for different input-output computations (see Discussion).

HAN Membrane Currents Triggered by OHN Activity

The main aim of our study was to reveal system-level relations between OH input and HAN output. We assume that HAN firing output is triggered by membrane currents evoked by coreleased OHN transmitters (which generate firing patterns after a further series of interactions with biophysical and geometric properties of the HAN membrane; Haas and Reiner, 1988). To confirm existence of these currents, we performed whole-cell voltage-clamp recordings from HANs. As expected from OHR2s present on HANs (Eriksson et al., 2001; Willie et al., 2003), OHN stimulation generated a significant progressive inward shift in the baseline current (Figures 3A and 3B). The size of this current was the same order of magnitude to currents evoked by a midnanomolar concentration of bath-applied OH peptide (Figure 3B), possibly providing a rough estimate of peptide levels arising from intrinsic peptide release. The inward current shift was significant both in control conditions (Figure 3B) and in CNQX (2.2 ± 0.6 pA, $n = 8$, $p < 0.01$) and was confirmed to require OHR2 by block with TCS (control + TCS: see Figure 3B; CNQX + TSC: 0.8 ± 0.7 pA, $n = 7$, $p > 0.2$). The TCS-sensitive shift in inward current upon OHN stimulation was not significantly different between control and CNQX conditions ($p > 0.05$ in unpaired t test).

Mirroring the failure of glutamate to transmit a sustained firing output (Figure 2A), there was a progressive falloff in glutamatergic currents (postsynaptic currents [PSCs]) during OHN stimulation (note that CNQX-sensitive photostimulated PSCs were not blocked by TCS, which was present throughout; Figures 3C–3F). This falloff was seen in both the total excitatory PSC frequency (Figure 3C) and in PSC success (i.e., increased failure of flashes to evoke PSCs; Figure 3D). The amplitude of optically evoked glutamatergic PSCs also tended to fall slightly with prolonged OHN stimulation (Figures 3E and 3F). Disappearance of the OHN→HAN^{AMPAR} current response during prolonged steady OHN stimulation was not due to irreversible vesicle depletion, because in all cells tested, the response was seen again after a 1–2 min "rest" without OHN stimulation ($n = 40$ cells).

DISCUSSION

Our results quantify the roles of OHN excitatory cotransmitters in input-output operation of a key arousal-controlling module in the brain. A central observation is that OH and glutamate convert the same OHN input into strikingly different temporal patterns of HAN spiking. These two spike responses could coexist in the same postsynaptic cell (Figures 1A and 1B). OH cotransmission was required for sustaining the postsynaptic firing responses to OHN activity for physiologically relevant durations. This provides direct evidence that endogenous OH release mediates spike transfer between brain circuits. In fact, under some conditions, OH generated more spikes than coreleased glutamate (Figure 1D).

We found that OH transmission required a higher presynaptic activity than glutamate transmission, corroborating previous inferences from less specific stimulation (Dutton and Dyball, 1979; Lundberg et al., 1986; Schöne and Burdakov, 2012; van den Pol, 2012; Verhage et al., 1991). At the level of spike output of the OHN→HAN circuit, we found little evidence for interactions between OH and glutamate, as implied by pharmacological independence of the two outputs across stimulation intensities and durations. This is surprising, because exogenously applied OH peptides can modulate glutamate transmission in other circuits (Lambe and Aghajanian, 2003; van den Pol et al., 1998). Perhaps OH-glutamate synergies depend on presynaptic OHRs, which are differentially expressed and/or activated in different circuits and at different levels of neural activity.

The glutamate firing response rose and fell rapidly, while OH firing response escalated linearly during unchanging OHN stimulation of physiological duration. If these differences reflected the cutting of OHN axons in our preparation, we would expect the reverse, i.e., OH transmission depleting rapidly, because glutamate is made in the terminals but peptides are made in the soma (Bear et al., 2001). The glutamate rundown was reversible in the same cell after a stimulation break, suggesting functional recycling. It is tempting to speculate that glutamate output decay during unchanging input could help detection of subsequent input changes, similar to the role of adaptation in sensory neurons tracking external stimuli (Carpenter, 2003).

Based on our data, the roles of glutamate and OH may be viewed as extracting and encoding, in HAN output, two distinct features of OHN input (Figure 4A). From this viewpoint, the transient AMPAR signaling may be seen as rapidly communicating changes or trends in OHN activity (e.g., analogously to a derivative controller; DiStefano et al., 2012). Conversely, OHR2-dependent operation (i.e., linear increase in output during constant input) may function as an integral controller whose output is proportional to integrated input (DiStefano et al., 2012). Interestingly, when placed in a feedback loop, integral controllers provide the key operation required for stable output in diverse systems (Aström and Hagglund, 1995; Csete and Doyle, 2002; DiStefano et al., 2012; Yi et al., 2000). Many existing lines of experimental evidence argue that the OHN→HAN module stabilizes wakefulness and may be considered a part of a feedback loop (e.g., via a hypothetical arrangement in Figure 4B). It may therefore be important to investigate whether OH peptides implement some form of integral control in the brain.

In summary, our data show that fast and slow cotransmitters can convert OHN activity into parallel and nonredundant spike streams in the same postsynaptic neuron. This clarifies functional benefits of OH cotransmission for diversifying neural circuit performance and computation and offers a cybernetic framework for reverse engineering pathophysiological OHR2 signaling.

EXPERIMENTAL PROCEDURES

Animal procedures followed United Kingdom Home Office regulations. ChR2 was introduced into OHNs using cre-dependent viruses in orexin-cre mice (Schöne et al., 2012; Matsuki et al., 2009). Effects of OH transmission were isolated by blocking glutamate and GABA transmission and confirmed as requiring OH receptors by blockade with 10 μM TCS-OX2-29 ("TCS," an OHR2 receptor blocker; Huang et al., 2010; Smart et al., 2001; Xiao et al., 2013) and/or 10 μM SB-334867 ("SB," an OHR1 blocker at this concentration;

OPEN
ACCESS
CellPress

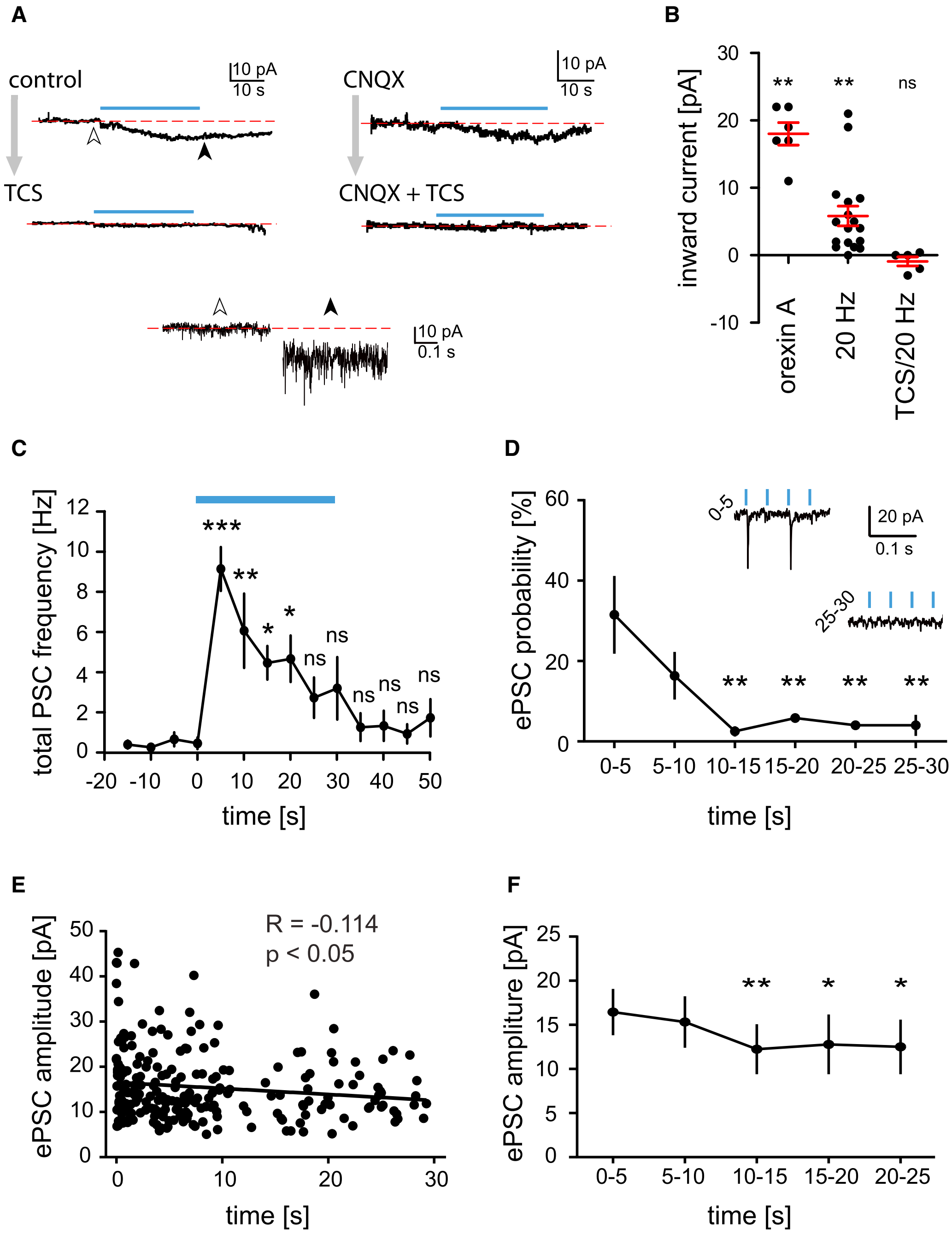

Figure 3. HAN Membrane Currents Evoked by OH and Glutamate Inputs from OHN

(A) Examples of slowly developing shifts in somatically recorded whole-cell current (at −70 mV) induced by 20 Hz OHN stimulation (blue bars) (n = 6 and 7 cells for control and CNQX groups, respectively). Top four traces are low-pass filtered for visual clarity. Bottom two traces are expansions at points indicated by arrowheads on top left trace.

(B) Inward shift in current baseline induced by bath-applied OH (300 nM) and by the optical stimulation (as in A) with and without TCS. Data are means ± SEM.

(C) Time course of total excitatory PSCs (inward currents at −70 mV) during 30 s 20 Hz optical stimulation recorded in TCS (significance relative to −15 to −20 s bin, n = 3 cells). Data are means ± SEM.

(D) Success rate for converting flashes to glutamate PSC (significance relative to 0–5 s bin) in the same data set as in (C). Data are mean ± SEM.

(E) Amplitudes of optically evoked PSCs during 20 Hz 30 s stimulation, recorded in TCS.

(F) Average amplitudes of optically evoked PSCs in different time bins (significance relative to 0–5 s bin, and same data set as in E). Data are means ± SEM.

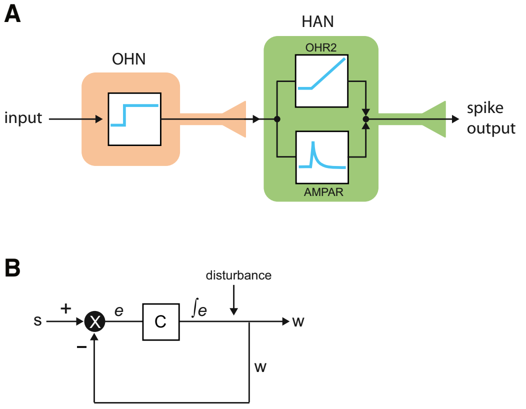

Figure 4. Model for Functional Logic of OH-Glutamate Co-transmission

(A) Cartoon of OHN→HAN circuit, overlayed with a theoretical engineering scheme, viewing OHR2s and AMPARs as a control module generating integral-derivative-like signals.

(B) A canonical integral feedback loop (simplified from Csete and Doyle, 2002; Aström and Hagglund, 1995). Integration in C compensates for disturbances to output w, allowing w to follow s despite disturbance (Aström and Hagglund, 1995; DiStefano et al., 2012). Hypothetically, to protect arousal signals (w) from instability, C could correspond to OHR2-expressing cells (e.g., HANs) and e could come from OHNs driven by positive inputs s (e.g., sounds; Mileykovskiy et al., 2005) and negative-feedback inputs w (e.g., serotonin, Li et al., 2002). The intermittent, disturbance-associated firing of OHN in vivo (Mileykovskiy et al., 2005) is consistent with this position of OHNs in the feedback loop. Note that integral (but not proportional or derivative) transformation of e by C is necessary and sufficient for accurate and disturbance-resistant tracking of s by w (Aström and Hagglund, 1995). Indeed, when OH or OHR2 is knocked out, OHNs cannot stabilize wakefulness (Chemelli et al., 1999; Lin et al., 1999; Willie et al., 2003).

Smart et al., 2001). See Supplemental Experimental Procedures for further detail.

SUPPLEMENTAL INFORMATION

Supplemental Information includes Supplemental Experimental Procedures and one figure and can be found with this article online at http://dx.doi.org/10.1016/j.celrep.2014.03.055.

ACKNOWLEDGMENTS

This work was supported by grants from the European Research Council (D.B., ref. 200500), UK Medical Research Council (D.B., ref. MC_UP_1202/2), and Human Frontiers Science Foundation (D.B. and A.A., ref. RGY0076/2012). We thank Lauren Mulholland for linguistic improvements to the text.

Received: June 29, 2013
Revised: February 5, 2014
Accepted: March 13, 2014
Published: April 24, 2014

REFERENCES

Adamantidis, A.R., Zhang, F., Aravanis, A.M., Deisseroth, K., and de Lecea, L. (2007). Neural substrates of awakening probed with optogenetic control of hypocretin neurons. Nature *450*, 420–424.

Aström, K., and Hagglund, T. (1995). PID Controllers: Theory, Design, and Tuning (Research Triangle Park: Instrument Society of America).

Bear, M.F., Connors, B.W., and Paradiso, M.A. (2001). Neuroscience: Exploring the Brain (Philadelphia: Lippincott Williams & Wilkins).

Burbach, J.P. (2011). What are neuropeptides? Methods Mol. Biol. *789*, 1–36.

Burnstock, G. (2004). Cotransmission. Curr. Opin. Pharmacol. *4*, 47–52.

Carpenter, R. (2003). Neurophysiology, Fourth Edition (London: Arnold).

Chemelli, R.M., Willie, J.T., Sinton, C.M., Elmquist, J.K., Scammell, T., Lee, C., Richardson, J.A., Williams, S.C., Xiong, Y., Kisanuki, Y., et al. (1999). Narcolepsy in orexin knockout mice: molecular genetics of sleep regulation. Cell *98*, 437–451.

Crocker, A., España, R.A., Papadopoulou, M., Saper, C.B., Faraco, J., Sakurai, T., Honda, M., Mignot, E., and Scammell, T.E. (2005). Concomitant loss of dynorphin, NARP, and orexin in narcolepsy. Neurology *65*, 1184–1188.

Csete, M.E., and Doyle, J.C. (2002). Reverse engineering of biological complexity. Science *295*, 1664–1669.

de Lecea, L., Kilduff, T.S., Peyron, C., Gao, X., Foye, P.E., Danielson, P.E., Fukuhara, C., Battenberg, E.L., Gautvik, V.T., Bartlett, F.S., 2nd., et al. (1998). The hypocretins: hypothalamus-specific peptides with neuroexcitatory activity. Proc. Natl. Acad. Sci. USA *95*, 322–327.

de Lecea, L., Jones, B.E., Boutrel, B., Borgland, S.L., Nishino, S., Bubser, M., and DiLeone, R. (2006). Addiction and arousal: alternative roles of hypothalamic peptides. J. Neurosci. *26*, 10372–10375.

DiStefano, J., Stubberud, A., and Williams, I. (2012). Feedback and Control Systems, Second Edition (New York: McGraw-Hill Education).

Dutton, A., and Dyball, R.E. (1979). Phasic firing enhances vasopressin release from the rat neurohypophysis. J. Physiol. *290*, 433–440.

Eriksson, K.S., Sergeeva, O., Brown, R.E., and Haas, H.L. (2001). Orexin/hypocretin excites the histaminergic neurons of the tuberomammillary nucleus. J. Neurosci. *21*, 9273–9279.

Furutani, N., Hondo, M., Kageyama, H., Tsujino, N., Mieda, M., Yanagisawa, M., Shioda, S., and Sakurai, T. (2013). Neurotensin co-expressed in orexin-producing neurons in the lateral hypothalamus plays an important role in regulation of sleep/wakefulness states. PLoS ONE *8*, e62391.

Haas, H.L., and Reiner, P.B. (1988). Membrane properties of histaminergic tuberomammillary neurones of the rat hypothalamus in vitro. J. Physiol. *399*, 633–646.

Haas, H.L., Sergeeva, O.A., and Selbach, O. (2008). Histamine in the nervous system. Physiol. Rev. *88*, 1183–1241.

Haass, M., Cheng, B., Richardt, G., Lang, R.E., and Schömig, A. (1989). Characterization and presynaptic modulation of stimulation-evoked exocytotic co-release of noradrenaline and neuropeptide Y in guinea pig heart. Naunyn Schmiedebergs Arch. Pharmacol. *339*, 71–78.

Hara, J., Beuckmann, C.T., Nambu, T., Willie, J.T., Chemelli, R.M., Sinton, C.M., Sugiyama, F., Yagami, K., Goto, K., Yanagisawa, M., and Sakurai, T. (2001). Genetic ablation of orexin neurons in mice results in narcolepsy, hypophagia, and obesity. Neuron *30*, 345–354.

Hirose, M., Egashira, S., Goto, Y., Hashihayata, T., Ohtake, N., Iwaasa, H., Hata, M., Fukami, T., Kanatani, A., and Yamada, K. (2003). N-acyl 6,7-dimethoxy-1,2,3,4-tetrahydroisoquinoline: the first orexin-2 receptor selective non-peptidic antagonist. Bioorg. Med. Chem. Lett. *13*, 4497–4499.

Huang, S.C., Dai, Y.W., Lee, Y.H., Chiou, L.C., and Hwang, L.L. (2010). Orexins depolarize rostral ventrolateral medulla neurons and increase arterial pressure and heart rate in rats mainly via orexin 2 receptors. J. Pharmacol. Exp. Ther. *334*, 522–529.

Lambe, E.K., and Aghajanian, G.K. (2003). Hypocretin (orexin) induces calcium transients in single spines postsynaptic to identified thalamocortical boutons in prefrontal slice. Neuron *40*, 139–150.

Lee, M.G., Hassani, O.K., and Jones, B.E. (2005). Discharge of identified orexin/hypocretin neurons across the sleep-waking cycle. J. Neurosci. *25*, 6716–6720.

Li, Y., Gao, X.B., Sakurai, T., and van den Pol, A.N. (2002). Hypocretin/Orexin excites hypocretin neurons via a local glutamate neuron-A potential

mechanism for orchestrating the hypothalamic arousal system. Neuron *36*, 1169–1181.

Lin, L., Faraco, J., Li, R., Kadotani, H., Rogers, W., Lin, X., Qiu, X., de Jong, P.J., Nishino, S., and Mignot, E. (1999). The sleep disorder canine narcolepsy is caused by a mutation in the hypocretin (orexin) receptor 2 gene. Cell *98*, 365–376.

Lundberg, J.M., Rudehill, A., Sollevi, A., Theodorsson-Norheim, E., and Hamberger, B. (1986). Frequency- and reserpine-dependent chemical coding of sympathetic transmission: differential release of noradrenaline and neuropeptide Y from pig spleen. Neurosci. Lett. *63*, 96–100.

Ma, X., Zubcevic, L., Brüning, J.C., Ashcroft, F.M., and Burdakov, D. (2007). Electrical inhibition of identified anorexigenic POMC neurons by orexin/hypocretin. J. Neurosci. *27*, 1529–1533.

Matsuki, T., Nomiyama, M., Takahira, H., Hirashima, N., Kunita, S., Takahashi, S., Yagami, K., Kilduff, T.S., Bettler, B., Yanagisawa, M., and Sakurai, T. (2009). Selective loss of GABA(B) receptors in orexin-producing neurons results in disrupted sleep/wakefulness architecture. Proc. Natl. Acad. Sci. USA *106*, 4459–4464.

Mignot, E., Taheri, S., and Nishino, S. (2002). Sleeping with the hypothalamus: emerging therapeutic targets for sleep disorders. Nat. Neurosci. Suppl. *5*, 1071–1075.

Mileykovskiy, B.Y., Kiyashchenko, L.I., and Siegel, J.M. (2005). Behavioral correlates of activity in identified hypocretin/orexin neurons. Neuron *46*, 787–798.

Mochizuki, T., Arrigoni, E., Marcus, J.N., Clark, E.L., Yamamoto, M., Honer, M., Borroni, E., Lowell, B.B., Elmquist, J.K., and Scammell, T.E. (2011). Orexin receptor 2 expression in the posterior hypothalamus rescues sleepiness in narcoleptic mice. Proc. Natl. Acad. Sci. USA *108*, 4471–4476.

Petreanu, L., Huber, D., Sobczyk, A., and Svoboda, K. (2007). Channelrhodopsin-2-assisted circuit mapping of long-range callosal projections. Nat. Neurosci. *10*, 663–668.

Peyron, C., Tighe, D.K., van den Pol, A.N., de Lecea, L., Heller, H.C., Sutcliffe, J.G., and Kilduff, T.S. (1998). Neurons containing hypocretin (orexin) project to multiple neuronal systems. J. Neurosci. *18*, 9996–10015.

Peyron, C., Faraco, J., Rogers, W., Ripley, B., Overeem, S., Charnay, Y., Nevsimalova, S., Aldrich, M., Reynolds, D., Albin, R., et al. (2000). A mutation in a case of early onset narcolepsy and a generalized absence of hypocretin peptides in human narcoleptic brains. Nat. Med. *6*, 991–997.

Ren, J., Qin, C., Hu, F., Tan, J., Qiu, L., Zhao, S., Feng, G., and Luo, M. (2011). Habenula “cholinergic” neurons co-release glutamate and acetylcholine and activate postsynaptic neurons via distinct transmission modes. Neuron *69*, 445–452.

Ripley, B., Fujiki, N., Okura, M., Mignot, E., and Nishino, S. (2001). Hypocretin levels in sporadic and familial cases of canine narcolepsy. Neurobiol. Dis. *8*, 525–534.

Sakurai, T. (2007). The neural circuit of orexin (hypocretin): maintaining sleep and wakefulness. Nat. Rev. Neurosci. *8*, 171–181.

Sakurai, T., Amemiya, A., Ishii, M., Matsuzaki, I., Chemelli, R.M., Tanaka, H., Williams, S.C., Richardson, J.A., Kozlowski, G.P., Wilson, S., et al. (1998). Orexins and orexin receptors: a family of hypothalamic neuropeptides and G protein-coupled receptors that regulate feeding behavior. Cell *92*, 573–585.

Salio, C., Lossi, L., Ferrini, F., and Merighi, A. (2006). Neuropeptides as synaptic transmitters. Cell Tissue Res. *326*, 583–598.

Schöne, C., and Burdakov, D. (2012). Glutamate and GABA as rapid effectors of hypothalamic “peptidergic” neurons. Front. Behav. Neurosci. *6*, 81.

Schöne, C., Cao, Z.F., Apergis-Schoute, J., Adamantidis, A., Sakurai, T., and Burdakov, D. (2012). Optogenetic probing of fast glutamatergic transmission from hypocretin/orexin to histamine neurons in situ. J. Neurosci. *32*, 12437–12443.

Smart, D., Sabido-David, C., Brough, S.J., Jewitt, F., Johns, A., Porter, R.A., and Jerman, J.C. (2001). SB-334867-A: the first selective orexin-1 receptor antagonist. Br. J. Pharmacol. *132*, 1179–1182.

Thannickal, T.C., Moore, R.Y., Nienhuis, R., Ramanathan, L., Gulyani, S., Aldrich, M., Cornford, M., and Siegel, J.M. (2000). Reduced number of hypocretin neurons in human narcolepsy. Neuron *27*, 469–474.

van den Pol, A.N. (2003). Weighing the role of hypothalamic feeding neurotransmitters. Neuron *40*, 1059–1061.

van den Pol, A.N. (2012). Neuropeptide transmission in brain circuits. Neuron *76*, 98–115.

van den Pol, A.N., Gao, X.B., Obrietan, K., Kilduff, T.S., and Belousov, A.B. (1998). Presynaptic and postsynaptic actions and modulation of neuroendocrine neurons by a new hypothalamic peptide, hypocretin/orexin. J. Neurosci. *18*, 7962–7971.

Verhage, M., McMahon, H.T., Ghijsen, W.E., Boomsma, F., Scholten, G., Wiegant, V.M., and Nicholls, D.G. (1991). Differential release of amino acids, neuropeptides, and catecholamines from isolated nerve terminals. Neuron *6*, 517–524.

Williams, R.H., Jensen, L.T., Verkhratsky, A., Fugger, L., and Burdakov, D. (2007). Control of hypothalamic orexin neurons by acid and CO2. Proc. Natl. Acad. Sci. USA *104*, 10685–10690.

Williams, R.H., Alexopoulos, H., Jensen, L.T., Fugger, L., and Burdakov, D. (2008). Adaptive sugar sensors in hypothalamic feeding circuits. Proc. Natl. Acad. Sci. USA *105*, 11975–11980.

Willie, J.T., Chemelli, R.M., Sinton, C.M., Tokita, S., Williams, S.C., Kisanuki, Y.Y., Marcus, J.N., Lee, C., Elmquist, J.K., Kohlmeier, K.A., et al. (2003). Distinct narcolepsy syndromes in Orexin receptor-2 and Orexin null mice: molecular genetic dissection of Non-REM and REM sleep regulatory processes. Neuron *38*, 715–730.

Winsky-Sommerer, R., Yamanaka, A., Diano, S., Borok, E., Roberts, A.J., Sakurai, T., Kilduff, T.S., Horvath, T.L., and de Lecea, L. (2004). Interaction between the corticotropin-releasing factor system and hypocretins (orexins): a novel circuit mediating stress response. J. Neurosci. *24*, 11439–11448.

Woldan-Tambor, A., Biegańska, K., Wiktorowska-Owczarek, A., and Zawilska, J.B. (2011). Activation of orexin/hypocretin type 1 receptors stimulates cAMP synthesis in primary cultures of rat astrocytes. Pharmacol. Rep. *63*, 717–723.

Xiao, F., Jiang, M., Du, D., Xia, C., Wang, J., Cao, Y., Shen, L., and Zhu, D. (2013). Orexin A regulates cardiovascular responses in stress-induced hypertensive rats. Neuropharmacology *67*, 16–24.

Yamanaka, A., Tsujino, N., Funahashi, H., Honda, K., Guan, J.L., Wang, Q.P., Tominaga, M., Goto, K., Shioda, S., and Sakurai, T. (2002). Orexins activate histaminergic neurons via the orexin 2 receptor. Biochem. Biophys. Res. Commun. *290*, 1237–1245.

Yi, T.M., Huang, Y., Simon, M.I., and Doyle, J. (2000). Robust perfect adaptation in bacterial chemotaxis through integral feedback control. Proc. Natl. Acad. Sci. USA *97*, 4649–4653.

Yizhar, O., Fenno, L.E., Davidson, T.J., Mogri, M., and Deisseroth, K. (2011). Optogenetics in neural systems. Neuron *71*, 9–34.

Cell Reports, Volume *7*
Supplemental Information

Coreleased Orexin and Glutamate Evoke Nonredundant Spike Outputs and Computations in Histamine Neurons

Cornelia Schöne, John Apergis-Schoute, Takeshi Sakurai, Antoine Adamantidis, and Denis Burdakov

SUPPLEMENTARY MATERIALS AND METHODS

1. Genetic targeting and viral transduction

Animal procedures were in accordance with UK Home Office regulations. Expression of ChR2 in OHNs was carried out and functionally confirmed as illustrated in Fig. S1. Briefly, high-titer ($1.5*10^{12}$ vg/ml) adeno-associated viral (AAV) constructs rAAV2/1.EF1a-DIO-hChR2(H134R)-EYFP.WPRE.hGH (Addgene #20298; packaged at Vector Core, University of Pennsylvania) were stereotaxically injected into brains of orexin-Cre mice, bred in het-WT breeding pairs with C57BL/6 mice. 4-6 week-old male and female mice were anesthetized with isoflourane and head-fixed in a stereotaxic frame (David Kopf Instruments). In some experiments in Fig. 3A,B, we also used rAAV2/Ef1a-DIO-ChR2(E123T/T159C)-mcherry ($3*10^{12}$ vg/ml; Addgene #35510 packaged at Vector Core, University of Pennsylvania) with similar results (data from two AAVs were pooled in this figure).

A borosilicate glass pipette tip (20-40 μm diameter) was stereotaxically lowered into the lateral hypothalamus. Three injections (each 50 nl, delivered at 75 nl/min) were made into LH in each hemisphere (bregma: -1.3 to -1.4 mm; midline ±0.9 mm; dorsal surface: -5.30, -5.15, and -5.00 mm). The pipette was gently withdrawn 6 min after final injection. Injections of identical AAV constructs into the lateral hypothalamus of WT mice did not generate ChR2-eYFP expression in the lateral hypothalamus (n = 3 mice), confirming that ChR2 expression was specific to Cre-containing cells.

2. Immunolabelling

We tested for specificity of transgene expression in orexin-Cre mice using immunohistochemistry. Orexin-Cre mice that had been previously injected with AAV containing ChR2-eYFP were terminally anesthetized and transcardially perfused with phosphate buffered saline containing 4 % PFA. Following postfixation brains were sectioned into 50 μm thick slices using a cryostat. Slices were stained using rabbit anti orexin-A (Phoenix Pharmaceutics, Inc., USA; 1:250) and mouse anti Cre-recombinase (Millipore, USA; 1:500) antibodies. We used goat anti rabbit alexa 647 (1:500) and goat anti mouse alexa 488 (1:1000) from Invitrogen for fluorescent labelling.

To label HANs during whole-cell recordings, these were filled with 0.2 % biocytin (Tocris) that was added to the intracellular recording solution. Cells were kept in the whole-cell mode for a minimum of 20 min. After recovery for at least 20 min, the tissue was fixed in 4 % PBS overnight. Biocytin filled cells were labeled with rabbit anti adenosine deaminase (Chemicon; 1:250). Immunofluorescence was achieved using goat anti-rabbit Alexa 555 (Invitrogen; 1:1000) antibodies and streptavidin tagged with Cy2 (Invitrogen; 1:1000).

Images were taken using an Olympus BX61WI confocal microscope (Olympus FluoView v 2.1b software) in a dynamic range of 16 bit using a 25x water immersion objective (NA 1.05, Olympus). Cy2 was excited with an argon laser at 488 nm, and its fluorescence collected between 570 and 670 nm using a spectral detector (Olympus). Alexa 555 was excited with a diode-pumped solid-state (DPSS) laser at 559 nm, and fluorescence emission collected at 570-670 nm using a spectral detector (Olympus).

When more than one fluorophore was detected, the sequential "between-lines" scanning mode of the microscope was used to achieve optimal separation of fluorescent signals.

3. Photostimulation and electrophysiology

Coronal slices containing the tuberomammillary hypothalamic nucleus (TMN) were made ≈2 months post-injection. 250 μm thick slices were cut with a Leica VT 1200S vibratome in ice-cold ACSF (see below), and allowed to recover for 1 hour at 35 °C in ACSF before recordings. For ChR2 stimulation with blue light, we used a LAMBDA DG-4 fast beam switcher (Sutter Instruments) with a Xenon lamp and ET470/40 nm band pass filter. Flashes of light (5 ms duration, ~10 mW/mm^2 power) were delivered onto ChR2-containing axons around the recorded cell via a 40x objective with 0.8 NA. Patch pipettes were manufactured from borosilicate glass, and their tip resistances were 4-6 MΩ when filled with K-gluconate solution (see below). Whole-cell recordings were carried out at 37 °C using an EPC-10 amplifier and PatchMaster software (HEKA Elektronik, Germany). Orexin/hypocretin neurons (identified by eYFP fluorescence and confirmed by electrical fingerprinting) routinely followed long trains of light flashes with time-locked trains of action potentials (Fig. S1A). Histamine neurons in the ventral TMN were unambiguously identified using classical electrophysiological, morphological, and post-immunostaining criteria (described with full references to original papers in Schöne et al., J Neurosci 2012, 32: 12437-12443). To dissect contributions of orexin/hypocretin and glutamate, we compared histamine neuron responses with and without pharmacological blockers of different neurotransmitter receptors. Only cells with access resistances of <20 MΩ were used for analysis. Current signals were low-pass filtered at 3 kHz and digitized

at 10 kHz. Glutamate EPSCs were identified and analyzed using standard Minianalysis software (Synaptosoft). Only histamine neurons that showed fast synaptic responses to orexin/hypocretin neuron optical stimulation were used for further analysis.

Control experiments revealed that basal tone (without optical stimulation) of orexin/hypocretin or glutamate was too low to drive histamine neuron firing. Specifically, blocking OH2Rs with TCS did not alter spontaneous histamine neuron firing, as analyzed by examining 10 s intervals preceding optical stimulation (control: 1.7±1.0 Hz, TCS: 1.6±0.7 Hz, n=4 cells, p>0.5 by paired t-test). TCS also did not significantly shift baseline current at -70 mV (control: -38.36±7.1 pA, TCS: -36.4±9.1 pA, n=5 cells, p>0.5 by paired t-test). Similarly, blocking OH2Rs+OH1Rs (with TCS+SB, see Methods) did not alter spontaneous histamine neuron firing (control: 2.2±1.0 Hz, TCS+SB: 2±0.4 Hz, n=6 cells, p>0.5 by paired t-test). Finally, blocking AMPAR-mediated glutamate transmission with CNQX did not alter spontaneous histamine neuron firing (control: 2.7±0.7 Hz, CNQX: 2.4±0.8 Hz, n=6 cells, p>0.3 by paired t-test). CNQX also did not significantly shift baseline current at -70 mV (control: -25.8±5.7 pA, TCS: -23.7±3.6 pA, n=5 cells, p>0.4 by paired t-test).

4. Analysis

Data were analysed with Minianalysis (Synaptosoft), Matlab (Mathworks) or Prism (Graphpad). Histamine neuron firing responses to optical orexin neuron stimulation were separated from baseline firing by subtracting the average baseline firing during 10 s prior to stimulation in each cell. In group data plots, results are presented as means ± sem of multiple experimental trials (1-2 trials were recorded per cell), except

where stated otherwise. Student's unpaired or paired t-test, or one or two-way ANOVA followed by Bonferroni or Newman-Keuls posthoc tests, were used for statistical hypothesis testing; the significance labels in the figures are $*p < 0.05$, $**p < 0.01$, $***p < 0.001$, and $p > 0.05$ was taken as non-significant (ns).

5. Chemicals and solutions

ACSF was bubbled with 95% O_2 and 5% CO_2 and contained (in mM): 125 NaCl, 2.5 KCl, 2 $MgCl_2$, 2 $CaCl_2$, 1.2 NaH_2PO_4, 21 $NaHCO_3$, 1 d-(+)-glucose, 0.1 Na^+-pyruvate and 0.4 ascorbic acid. For whole-cell recordings, pipettes were filled with (in mM): 124 K-gluconate, 14 KCl, 10 HEPES, 1 EGTA, 5 MgATP, 0.3 Na_2GTP, 10 Na_2Phosphocreatine, pH = 7.3 with KOH. Orexin/hypocretin receptors were blocked with either 10 μM TCS-OX2-29 ("TCS", an OHR2 receptor blocker), and/or 10 μM SB-334867 ("SB", an OHR1 blocker at this concentration). AMPA glutamate receptors were blocked with 10 μM 6-cyano-7-nitroquinoxaline-2,3-dione (CNQX). NMDA glutamate receptors were blocked with 50 μM (2*R*)-amino-5-phosphonovaleric acid (AP5). $GABA_{A/C}$ receptors were blocked with 50 μM picrotoxin (PiTX). For bath application of orexin/hypocretin, we used 300 nM orexin-A from Bachem. All other chemicals were from Sigma or Tocris.

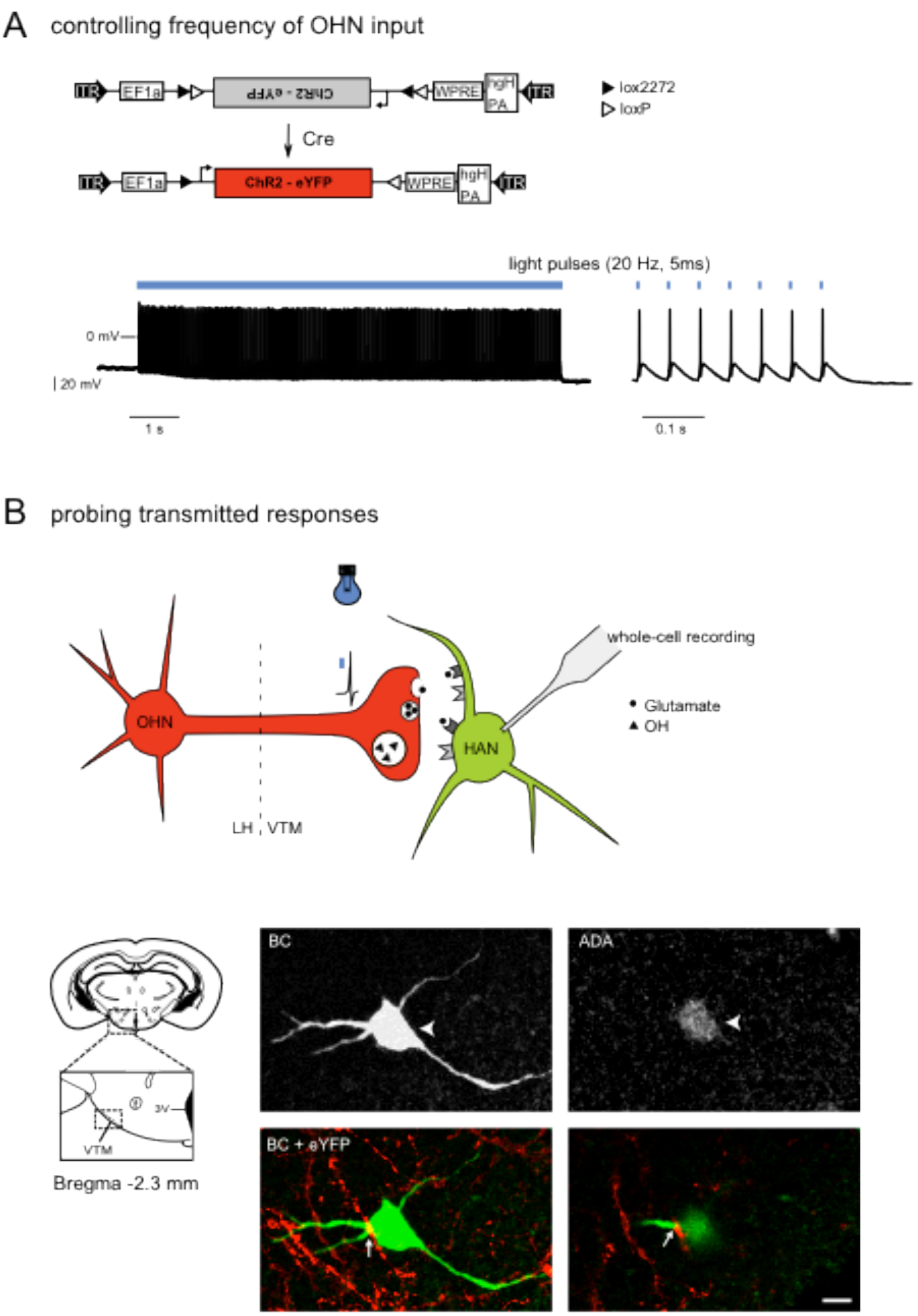

Figure S1. *Studying functional transmission in orexin → histamine microcircuit* (Illustration of methodological background to experiments in main Figures 1-3).

A. Schematic of virally-delivered constructs used to transduce orexin-Cre neurons (OHNs) with ChR2 (top), after which OHN spikes routinely followed optical stimuli in brain slices (bottom, typical example of n = 10 cells).

B: Schematic of experimental strategy for probing postsynaptic responses of histamine neurons (HANs) to OHN stimulation (top), and typical confocal images (bottom) of a HAN filled with biocytin (BC) and confirmed by post-recording immunocytochemistry to contain HAN marker adenosine deaminase (ADA, arrowheads, n = 3/3 cells). ChR2-eYFP fibers (shown in red pseudo-color) make close contact with HAN (arrows in bottom images). Scale bar, 10 µm.
